# Closing the loop: Benefits and challenges of sharing clinical trial results with participants after trial close-out

**DOI:** 10.1186/s12874-026-02787-3

**Published:** 2026-02-04

**Authors:** Jodi L. Gallant, Tristan Paranavitana, Sofia Bzovsky, Kaitlyn Pusztai, Paula McKay, Debra Marvel, Jeffrey L. Wells, Julie Menard, Jamal Al-Asiri, Joseph T. Patterson, Gerard Slobogean, Sheila Sprague, the PREP-IT Investigators, Mohit Bhandari, Anthony D. Harris, C. Daniel Mullins, Lehana Thabane, Amber Wood, Gregory J. Della Rocca, Joan Hebden, Kyle J. Jeray, Lucas S. Marchand, Lyndsay M. O’Hara, Robert Zura, Christopher Lee, Michael J. Gardner, Jenna Blasman, Jonah Davies, Stephen Liang, Monica Taljaard, P. J. Devereaux, Gordon H. Guyatt, Diane Heels-Ansdell, Jana Palmer, Jeff Friedrich, Nathan N. O’Hara, Frances Grissom, I. Leah Gitajn, Saam Morshed, Robert V. O’Toole, Bradley A. Petrisor, Franca Mossuto, Manjari G. Joshi, Jean-Claude D’Alleyrand, Justin Fowler, Jessica Rivera, Max Talbot, David Pogorzelski, Shannon Dodds, Jordan Leonard, Silvia Li, Alejandra Rojas, Gina Del Fabbro, Olivia Paige Szasz, Alexandra Minea, Kevin Murphy, Andrea Howe, Yasmin Degani, Haley Demyanovich, Michelle Medeiros, Genevieve Polk, Eric Kettering, Nirmen Mahal

**Affiliations:** 1 Department of Surgery, Hamilton Health Sciences, Hamilton, ON Canada; 2 Division of Orthopaedic Surgery, Department of Surgery, McMaster University, 175 Longwood Road S, Suite 210A, Hamilton, ON L8P 0A1 Canada; 3 Patient Representative, University of Maryland Baltimore, Baltimore, MD USA; 4 Research Centre of the Centre Hospitalier Universitaire de Sherbrooke, Sherbrooke, QC Canada; 5 Department of Orthopaedic Surgery, Keck School of Medicine of University of Southern California, Los Angeles, CA USA; 6 Center for Orthopaedic Injury Research and Innovation, Department of Orthopaedics, University of Maryland School of Medicine, R Adams Cowley Shock Trauma Center, Baltimore, MD USA; 7 Department of Health Research Methods, Evidence, and Impact, McMaster University, Hamilton, ON Canada

**Keywords:** patient engagement, orthopaedic trauma, randomized clinical trials (RCTs), open science, sharing results

## Abstract

**Background:**

Clinical trial participants have a right to know the results of the trials in which they participate. Trial results are often not shared directly with participants and concerns with privacy and resource constraints may prevent researchers from contacting participants after trial completion.

**Questions/Purposes:**

The objectives of this cross-sectional study were to explore the feasibility of contacting orthopaedic fracture trial participants after trial completion and to determine the preferences and priorities of the participants who wished to know the results.

**Patients/Methods:**

Following the publication of the primary manuscript, we attempted to contact participants from the completed PREPARE trial at Hamilton Health Sciences to determine if they would like to know the trial results. We asked participants about their preferences for receiving trial results, their experiences upon learning them, and if they wished to learn which treatment they received.

**Results:**

Twenty-eight percent (181/641) of PREPARE trial participants contacted agreed to participate in this study. We found that 95.5% (95% CI 91.0%—97.9%) of respondents wished to know the trial results and the preferred method was through viewing summary posters via an online link (78.2%; 95% CI 71.1%—84.0%). Most felt satisfied after learning the trial results (67.8%; 95% CI 59.5%- 75.2%) and 82.2% (95% CI 75.2%—87.5%) wanted to know which treatment they received. Fifty-one percent (95% CI 42.7%—58.7%) reported that learning the results increased their likelihood of participating in a future trial.

**Conclusions:**

Although it was challenging to both contact and re-engage participants after completing an orthopaedic trial that involved minimal participant burden, our study findings suggest that learning the trial results may have a positive impact on individual participants and the research community. Given the limited understanding of results among our respondents, researchers should have processes in place to engage participants meaningfully throughout the trial and proactively discuss with them how the results will be shared once the trial is complete.

**Level of evidence:**

IV.

**Supplementary Information:**

The online version contains supplementary material available at 10.1186/s12874-026-02787-3.

## Introduction

Clinical trial participants are often not directly offered the results of the trials in which they participate [1–11]. The Declaration of Helsinki states that participants should be offered the opportunity to learn the findings of clinical trials in which they participate [12]; however, the current literature indicates a gap between this principle and common practice with regards to results communication [1, 13]. A survey of participants in oncology trials found that only 7.9% of respondents could recall being informed of the clinical trial findings [1], even though a similar survey reported that 98.7% of oncology trial participants felt that participants should be offered the findings of their clinical trial [13]. Additionally, even when results are available to participants, they are often not delivered through active methods; rather, the participant has to make an effort to locate the information [4, 7, 9, 14–16]. An analysis of clinical trial applications between 2012 and 2017 in the UK found that although 87.7% of trials mentioned research dissemination, less than 19% of these trials included plans to use an active method of communication [14]. In Canada, the major federal health research funder provides a patient engagement framework for patient-oriented research; however, this framework document does not include specific information on sharing results with participants, and data on the prevalence of results sharing with participants in Canada is unavailable [17].

There is also conflicting evidence regarding the best methods by which to deliver trial results to participants. Email, telephone calls, and in-person meetings with the participant’s doctor have all been found to be the most preferred method of results communication in different papers [13, 18, 19]. The “Show RESPECT” study evaluated methods for sharing results and found high patient satisfaction after providing ovarian cancer trial participants with choices on whether to receive the results or not and on the method by which they receive the results [9].

Additionally, resource limitations, such as time and funding, may inhibit researchers from using certain approaches to share trials results with participants, especially in large, multisite trials [2]. From a feasibility perspective, the FOCUS trial researchers recently found sharing trial results by email to be feasible for a large, randomized trial that compared two treatments for stroke [20]. Several other studies report sharing trial summary results online as a low-cost option [4, 14, 19].

While research in these fields provides useful insights, the findings may not be generalizable to participants in orthopaedic fracture research as most fractures are successfully treated, and after treatment, are not perceived as inherently long-term or life-threatening conditions in the way that a stroke or cancer diagnosis may be. To address this gap in the literature, we conducted a cross-sectional study to explore the feasibility of contacting orthopaedic fracture trial participants or their substitute decision makers (SDMs) after trial completion to share the trial results with them. The secondary objectives of this study were to determine the: 1) proportion of participants or SDMs who wished to know the trial results; 2) preferred methods of communicating results; 3) reasons why participants or SDMs may not wish to hear the results of the trial; 4) impact of receiving the trial results on participants or SDMs; 5) participants’ or SDMs’ understanding of the trial results, and 6) proportion of participants or SDMs that wish to learn the treatment received.

## Patients/Methods

We conducted a cross-sectional survey of participants from the PREPARE trial at the Hamilton Health Sciences—General Hospital (HHS) in Ontario, Canada [21]. We obtained research ethics board approval from the Hamilton Integrated Research Ethics Board (HiREB) prior to initiating the study (HiREB #4913).

### PREPARE

The PREPARE trial was a multi-centre, cluster-randomized crossover trial comparing two different antiseptic solutions used before surgery for open or closed fractures in adults [21]. At the HHS site, 653 of 954 screened participants (68.4%) above the age of 18 were enrolled. At the time of consent, PREPARE participants were told that trial results would be available on ClinicalTrials.gov upon trial completion. Fewer surgical site infections were identified in closed fracture participants who received DuraPrep™ (2.4%) compared to those receiving ChloraPrep™ (3.3%), a difference which was statistically significant (*p* = 0.049) [21]. In open fracture participants, surgical site infections were reported in 6.5% of participants receiving DuraPrep™ and 7.3% of participants receiving ChloraPrep™ [21]. This difference was not statistically significant (*p* = 0.45).

### Participant screening

Once results for the PREPARE trial were made publicly available in February 2024, all 653 participants enrolled from HHS in the PREPARE trial were assessed for eligibility in this cross-sectional survey study. To be eligible, the respondent had to be a participant or SDM of a participant in the PREPARE trial at HHS and provide informed consent for participating in this follow-up study. We excluded participants who: 1) withdrew consent during their participation in the PREPARE trial or 2) passed away during the PREPARE trial.

### Informed consent

Approximately 24 to 48 months after participants had completed their participation in the PREPARE trial, research personnel sent an email to each eligible participant inviting them to participate in this follow-up study. The email included a link to the McMaster REDCap (Research Electronic Data Capture) system where the participant could review the consent form and provide informed consent. If a potential participant did not have an email address or did not respond, research personnel contacted them via telephone to obtain informed consent. If a participant could not provide electronic consent, research personnel mailed a paper copy of the informed consent form and called the participant to obtain verbal consent. If a participant had an SDM, the SDM provided informed consent on behalf of the PREPARE participant.

### Data collection

After providing informed consent to participate in this follow-up study, participants completed a questionnaire in the REDCap system asking whether they wished to know the trial results. Questions were adapted for the PREPARE trial population and based on the questionnaire used by the LOVIT Trial Investigators [22]. Participants who did not wish to learn the trial results were asked to provide a reason. Those who wished to know the trial results were asked questions regarding the emotional impact of the results, their understanding of the summarized results, and if they wished to know which treatment (antiseptic solution) they received. If a participant was unable to complete the questionnaire electronically in REDCap, research personnel completed the questionnaire with them over the telephone. Study data were collected using REDCap electronic data capture tools hosted at McMaster University. REDCap is a secure, web-based software platform designed to support data capture for research studies [23, 24].

### Sharing the summarized trial results

Participants were given the option of receiving a summary of the trial results either online via the REDCap system, verbally over a telephone call from research personnel, or by text message. Participants opting to receive results via REDCap or text message were presented with two posters summarizing the trial results and were provided with a link to the published manuscript (Appendix). Summary posters were designed in collaboration with patient partners to ensure content clarity. If participants opted to receive results verbally, research personnel contacted the participant by telephone, read a summary script of the results and answered any questions the participant had. All participants were contacted by one of two research team members who were well-trained and familiar with the PREPARE trial to ensure they were able to answer any questions raised by study participants.

### Sample size

This study used a sample of convenience by attempting to contact all the eligible participants enrolled in the PREPARE trial at HHS. This approach allowed us to explore the feasibility of contacting participants after trial completion and based on the percentage that responded, to estimate the percentage of PREPARE participants interested in learning the results.

### Data analysis

Results were summarized using descriptive statistics, reported as count (percent) for categorical variables with corresponding 95% confidence intervals (95% CIs). All analyses were conducted in R (version 4.3.2, R Foundation for Statistical Computing, Vienna, Austria).

## Results

### Participant identification

Of the 653 participants enrolled in the PREPARE trial at HHS, six withdrew consent to participate and six passed away prior to trial completion. One hundred and eighty-one (28.2%) of the remaining 641 PREPARE participants consented to participate in this follow-up study, and 146 of 181 (80.7%) participants completed the full questionnaire. Reasons for exclusion are listed in Table 1.

**Table 1:** Reasons for exclusion

	**N (%) *N* = 460**
Declined to participate	36 (7.8)
Did not respond to invitation to participate	389 (84.6)
No active contact information	31 (6.7)
Deceased (after participating in PREPARE)	4 (0.9)

### Learning the PREPARE trial results

**Fig. 1:**
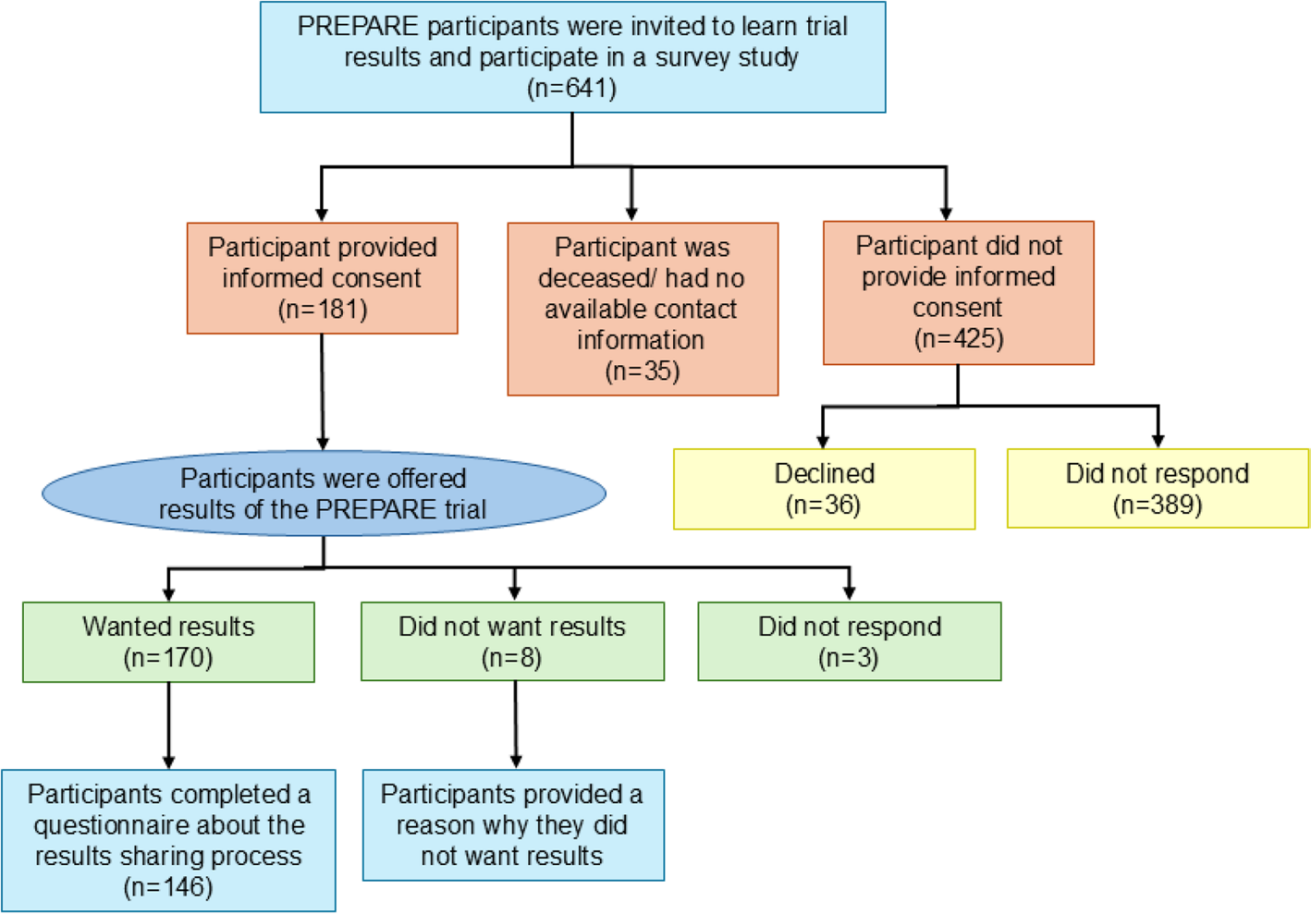
Participant Screening and Study Overview

One hundred and seventy (95.5%; 95% CI 91.0%—97.9%) participants indicated that they wished to learn the trial results, while eight (4.5%; 95% CI 2.3%—8.6%) participants did not wish to learn the results (Fig. 1). To note, three of the 181 participants (1.7%; 95% CI 0.6%—4.8%) who consented did not complete any of the questionnaire (Table 2). Of the eight participants who did not wish to learn the PREPARE trial results, six indicated not being interested (75.0%; 95% CI 35.6%—95.5%), one indicated that they did not feel that they would benefit from the trial results as a lay person (12.5%; 95% CI 0.6%—47.1%), and one stated being happy to take part in the trial in hopes that the research would help others (12.5%; 95% CI 0.6%—47.1%) (Table 2).

**Table 2:** Preferred method of obtaining results

	**N (%; 95% CI) *N* = 178**
Participants wanting to learn the results of the PREPARE trial, n (%)	
Yes	170 (95.5; 91.0, 97.9)
No	8 (4.5; 2.3, 8.6)
Preferred method of obtaining the results of the PREPARE trial, n (%)	*N* = 170
Online	133 (78.2; 71.1, 84.0)
Telephone	20 (11.8; 7.5, 17.8)
Text	17 (10.0; 6.1, 15.8)
Reasons participants did not wish to know the results of the PREPARE	*N* = 8
trial, n (%)	
Not interested	6 (75.0; 35.6, 95.5)
Other*	2 (25.0; 4.5, 64.4)

^*^Other: one participant indicated that they were not a specialist and trusted the results and one participant indicated they were happy to take part in the trial in hopes that the research would help others

### Preferred methods of sharing results

The majority (78.2%; 95% CI 71.1%—84.0%) of participants who wished to learn the PREPARE trial results indicated that their preferred method of obtaining results was online through a link to the results summary posters, followed by telephone (11.8%; 95% CI 7.5%—17.8%), and text message (10.0%; 95% CI 6.1%—15.8%) (Table 2). Most participants receiving the trial results by telephone (95%; 95% CI 71.9%—99.7), online (94%; 95% CI 87.4%—97.3%), and text (92%; 95% CI 59.8%—99.6%) felt either very satisfied or satisfied with the method by which they received the trial results (Table 3). Similarly, 84.2%% of those receiving results by telephone (95% CI 62.4—94.5%), 90.4% of those receiving results online (95% CI 83.7%—94.6%), and 91.7% of those receiving results by text (95% CI 64.6%—99.6%) did not suggest any other communication methods for sharing results in future (Table 3). The 10.3% (95% CI 6.3%—16.3%) with suggestions proposed mailing a paper copy of the results and media announcements.

**Table 3:** Method of communicating PREPARE trial results

	**Telephone N (%; 95% CI) *N* = 19**	**Online N (%; 95% CI) *N* = 115**	**Text N (%; 95% CI) *N* = 12**
Level of satisfaction with the method (telephone, online, or text) by which participant chose to learn the results of the			
PREPARE trial, n (%)			
Satisfied or very satisfied	18 (95; 71.9, 99.7)	108 (94; 87.4, 97.3)	11 (92; 59.8, 99.6)
No Preference	0 (0; 0.0, 20.9)	7 (6.1; 2.7, 12.6)	1 (8.3; 0.4, 40.2)
Dissatisfied or very dissatisfied	1 (5.3; 0.3, 28.1)	0 (0; 0.0, 4.0)	0 (0; 0.0, 40.2)
Would you suggest any other methods of communication for sharing the results of a clinical trial in the future?, n (%)			
Yes	3 (16; 4.2, 40.5)	11 (9.6; 5.1, 16.8)	1 (8.3; 0.4, 40.2)
No	16 (84.2; 62.4, 94.5)	104 (90.4; 83.7, 94.6)	11 (91.7; 64.6, 99.6)

### Emotional impact of the PREPARE trial results on participants

Participants were asked to select at least one of 13 possible emotional responses, including “I don’t know” and “Other, please describe”, to describe how they felt after learning the trial results. Most participants indicated that the PREPARE trial results made them feel satisfied [99/146 (67.8%; 95% CI 59.5%—75.2%)]. Others reported feeling indifferent (20.5%; 95% CI 14.5%—28.2%), empowered (6.2%; 95% CI 3.0%—11.7%), and proud (5.5%; 95% CI 2.6%—10.9%), as selected from a list of options. Nearly all respondents (95.9%; 95% CI 91.3%—98.1%) either agreed or strongly agreed that their participation in the PREPARE trial will help doctors make decisions about patient care in the future. Two participants (1.4%; 95% CI 0.4%—4.9%) agreed or strongly agreed that they wished to not have been informed of the trial results but did not specify why. Half of the participants (50.7%; 95% CI 42.7%—58.7%) indicated that they were now more likely to participate in future clinical trials, 47.3% (95% CI 39.3%—55.3%) indicated that the likelihood had not changed, and three individuals (2.1%; 95% CI 0.7%—5.9%) said that they were less likely to participate again (Table 4). Most participants either agreed or strongly agreed that researchers should: always share trial results with participants (93.2%; 95% CI 87.9%—96.2%), share trial results when a difference is found between the trial interventions (94.5%; 95% CI 89.6%—97.2%), and share trial results even when no difference is identified between trial interventions (91.1%; 95% CI 85.4%—94.7%) (Table 4).

**Table 4:** Emotional impact of the PREPARE Trial results on participants and importance of communicating research results

	**N (%; 95% CI) *N* = 146**
How results of the PREPARE trial made participants feel, n (%)*	
Satisfied	99 (67.8; 59.5, 75.2)
Indifferent	30 (20.5; 14.5, 28.2)
Empowered	9 (6.2; 3.0, 11.7)
Proud	8 (5.5; 2.6, 10.9)
Surprised	7 (4.8; 2.1, 10.0)
Don’t know	7 (4.8; 2.1, 10.0)
Other	7 (4.8; 2.1, 10.0)
Concerned	1 (0.7; 0.0, 4.3)
Nervous	1 (0.7; 0.0, 4.3)
Disappointed	1 (0.7; 0.0, 4.3)
I prefer not to answer	1 (0.7; 0.0, 4.3)
Upset	0 (0.0; 0.0, 3.2)
Guilty	0 (0.0; 0.0, 3.2)
Angry	0 (0.0; 0.0, 3.2)
I feel that my participation in the PREPARE trial will help doctors make decisions about patient care in the future, n (%)	
Agree or strongly agree	140 (95.9; 91.3, 98.1)
Neither agree nor disagree	6 (4.1; 1.7, 9.1)
Disagree or strongly disagree	0 (0.0; 0.0, 3.2)
Now that I know the PREPARE trial results, I wish that I had not been informed, n (%)	
Agree or strongly agree	2 (1.4; 0.4, 4.9)
Neither agree nor disagree	30 (20.5; 14.8, 27.8)
Disagree or strongly disagree	114 (78.1; 70.7, 84.0)
The likelihood that I would personally consent to participate in a clinical trial in the future is, n (%)	
Increased	74 (50.7; 42.7, 58.7)
Decreased	3 (2.1; 0.7, 5.9)
Not changed	69 (47.3; 39.3, 55.3)
Researchers should always inform participants of the results of their clinical trial, n (%)	
Agree or strongly agree	136 (93.2; 87.9, 96.2)
Neither agree nor disagree	10 (6.8; 3.8, 12.1)
Disagree or strongly disagree	0 (0.0; 0.0, 3.2)
It's important that researchers share the results of a clinical trial when there is a difference between the treatments being compared (e.g. if one of the cleaning solutions reduces the risk of infection), n (%)	
Agree or strongly agree	138 (94.5; 89.6, 97.2)
Neither agree nor disagree	8 (5.5; 2.6, 10.9)
Disagree or strongly disagree	0 (0.0; 0.0, 3.2)
It's important that researchers share the results of a trial even if there is NO difference between the treatments being compared (e.g. the cleaning solutions do not change the risk of infection), n (%)	
Agree or strongly agree	133 (91.1; 85.4, 94.7)
Neither agree nor disagree	7 (4.8; 2.1, 10.0)
Disagree or strongly disagree	6 (4.1; 1.7, 9.1)

^*^More than one response could be selected

### Understanding of the PREPARE trial results

Most respondents indicated that results were easy to understand (97.2%; 95% CI 93.1%—98.9%). However, when asked to select the correct interpretation of the trial results, most (62.1%; 95% CI 54.0%—69.6%) selected the incorrect option or were unsure (Table 5).

**Table 5:** Understanding of the PREPARE trial summary results

	N (%; 95% CI) *N* = 145*
The summary of the PREPARE trial results is easy to understand, n (%)	
True	141 (97.2; 93.1, 98.9)
False	1 (0.7; 0.0, 4.3)
I don’t know	3 (2.1, 0.7, 5.9)
Interpretation of the PREPARE trial results, n (%)	
Selected correct interpretation	55 (37.9; 30.4, 46.0)
Selected incorrect interpretation or unsure	90 (62.1; 54.0, 69.6)

^*^One participant did not respond to these questions

### Sharing the treatment received

Most participants agreed that researchers should offer participants the opportunity to learn the treatment they received (89.0%; 95% CI 82.9%—93.1%) and 82.2% (95% CI 75.2%—87.5%) of respondents wanted to learn which antiseptic solution that they personally received (Table 6).

**Table 6:** Sharing the treatment received

	**N (%; 95% CI) *N* = 146**
Researchers should offer participants the opportunity to learn the treatment they received	
Agree or strongly agree	130 (89.0; 82.9, 93.1)
Neither agree nor disagree	16 (11.0; 6.9, 17.1)
Disagree or strongly disagree	0 (0.0; 0.0, 3.2)
Would you like to know which group you were in (iodine povacrylex group or chlorohexidine gluconate group)?, n (%)	
Yes	120 (82.2; 75.2, 87.5)
No	26 (17.8; 12.5, 24.8)

## Discussion

Although we found that 95.5% of PREPARE trial participants who agreed to be in this study wished to learn the results of the PREPARE trial, our participation rate in this study was lower than anticipated. This finding is intriguing, since it is unknown how much of this low response rate reflects the percentage of participants who truly were uninterested in the trial results and how much reflects the logistical limitations to contacting research participants years after being involved in a low-burden trial. Notably, of the 425 participants who did not provide consent to participate, only 36 explicitly declined, while the remaining 389 did not respond to the study team’s contact attempts. Regardless, our study demonstrated some of the benefits and challenges of upholding the ethical imperative of sharing clinical trial results with orthopaedic fracture trial participants.

### Benefits

As previously discussed, researchers have an ethical duty to make the results of their trials available to participants [12]. In addition to fulfilling this duty, sharing the results of clinical trials may have worthwhile benefits to both individual researchers and the larger scientific community [7, 8, 25]. We found that half of respondents (50.7%) stated that they were more likely to participate in another clinical trial after receiving the PREPARE trial results, and only 2.1% indicated being less likely. Other researchers noted that disseminating clinical trial findings to participants could increase participation in future research [2, 6–9, 19, 25, 26], with one survey finding that 73% of participants who received summarised results were more willing to participate in future research [25]. These results suggest that sharing trial results with participants could potentially increase future participation in clinical trials which would help advance the medical field. This is particularly relevant in surgical trials as timely enrollment of participants remains one of the biggest barriers to clinical trial success [26]. Future research should assess actual clinical trial participation after receiving the results of a trial they previously participated in. There was also a high rate of satisfaction with the communication methods used in this study. Most participants (78.2%) preferred to view results online and 93.8% of all participants were satisfied with their choice of communication method, indicating that receiving the trial results was important to at least a portion of the PREPARE trial participants. The high level of patient satisfaction with receiving the results online further supports this as a cost-effective option for large trials [4, 14, 20].

### Challenges

Given our study design, there were some unique limitations. To abide by ethics regulations, PREPARE participants were required to sign a new consent form for this follow-up study that documented their willingness to be offered the results of the trial and to complete a survey about the experience afterwards. Some PREPARE participants may not have felt comfortable or been interested in signing another consent form for this study, especially those participants not wanting to receive trial results. Additionally, over 68% (440 of 641) of PREPARE trial participants did not respond to our contact attempts inviting them to participate in this study and to learn the trial results. This may be due to changes in contact information, not wishing to know the results, or being unwilling to participate in another study. Unfortunately, we were not able to capture reasons why PREPARE trial participants declined to participate in this study, which may have limited this study to those participants with a relatively higher level of research engagement. Having information from participants who did not respond could have potentially altered our findings on why people do not wish to receive a summary of trial findings. Another limitation was the amount of time that had passed between participating in the PREPARE trial and being offered the results, due to the time required for data analysis and results publication of a large, international trial. Some participants had been enrolled several years ago, while others had been enrolled more recently. Due to this variation in timing, and the fact that participating in the PREPARE trial did not change their treatment or require any long follow-up questionnaires, some patients may have forgotten about their trial participation. This limits our study, as people who could not fully remember the trial could have had a different level of engagement with the trial results than those who completely remembered the experience. Finally, it is worth noting that although we had hoped that most participants would respond to our initial email and therefore minimize the time and staff resources required to share the trial results, many participants did not respond which required research personnel to follow up by telephone (if a number was available) or email, which greatly increased the needed staff resources. Based on this experience, we have made several recommendations for researchers when considering how to share the results of their clinical trial with participants.

#### Consider the unique characteristics of the orthopaedic trauma population

Many previous studies have focused on trial participants in oncology clinical trials [1, 13, 27]. These studies have yielded specialized results, including one survey which found that over 95% of patient respondents in cancer trials strongly agreed that most participants wish to receive the findings of studies they were in [1, 13, 27]. However, variations in trial design, participant involvement, treatment, and patient prognosis between orthopaedic trauma and oncology patients may explain the differences in wishing to know clinical trial results. The pragmatic nature of the PREPARE trial design may also have contributed to our low response rate, as there was minimal burden on participants in this trial (e.g. no trial medications, routine clinical follow-up, no questionnaires). As many participants had recovered from their injury, they may have felt that the trial results would not be meaningful to them. This could potentially result in a lower proportion of participants wishing to receive the trial results. Differences in the rate of participants who wish to learn their treatment allocation may also vary depending on whether they experienced the primary outcome of surgical-site infection in the PREPARE trial.

Regarding emotional responses of respondents, most participants indicated that they felt satisfied (67.8%) after learning the PREPARE trial results. Other research has reported that some participants or SDMs experienced negative emotions, such as feeling upset, concerned, or increased anxiety upon receiving trial results [27, 28]. Williams et al. found that nearly 20% of participants felt either concerned, or satisfied and concerned, after learning the trial results, whereas our study found that only 0.7% of participants felt nervous or concerned [26]. This may reflect the acute nature of the conditions of the PREPARE trial participants as compared to participants with a chronic disease or ongoing illness, as many orthopaedic trauma participants recover from their injuries with minimal long-term impairments. Our findings offer more insight into the preferences of clinical trial participants in the orthopaedic trauma field. Participants in different clinical research fields likely have different experiences, and so learning research findings could inevitably evoke different emotions in participants.

#### Offer results through various methods and engage participants during the trial to ensure understanding of results

Our study supports the current literature that suggests there is not a single preferred medium for sharing results and it may be beneficial to offer several methods to maximize accessibility and participant preference [10, 13, 18, 19]. However, although most of our survey respondents were satisfied with their choice of communication method, when asked about the content, many participants did not correctly interpret the trial results. This may indicate an inconsistency between how participants prefer to receive trial results and the most effective method for communicating results. Additionally, although we involved patient partners in designing our summary posters and most participants reported them as being easy to understand, this gap in understanding may indicate a low level of participant engagement in either the PREPARE trial or this study. It remains unknown whether participants did not have a good understanding of the research question while in PREPARE or if they just did not expect to be asked about the trial years later. It is worth considering whether sharing the results might have been more impactful if participants had a clearer understanding of the trial's purpose and value during their initial participation phase. Finally, it’s possible that because the results of the PREPARE trial were different for the open and closed fracture groups, participants may have been unsure about which group they had been in and therefore which results applied to them. This possible confusion supports the suggestion from the Show RESPECT framework that personal discussion or personalized results may be necessary when trial results are complex [10].

#### Incorporate a plan to share results into initial trial development

Our study faced several barriers to contacting participants after their direct participation in the trial had ended. Specifically, a lack of reliable, up-to-date contact information, and participants having a limited memory of the trial or research team, made it difficult to engage participants in this follow-up study. Most participants in our study agreed that researchers should always share trial results with participants but due to the inherent delay between actively collecting participant data and having results available for public sharing, it can be challenging to have both current contact information and permission from the ethics board to contact past participants. To overcome this, researchers should engage their local ethics board during their initial trial submission to ensure approval of their plans to share trial results with participants and the communication methods to be used. This may include language on the informed consent form and questions on case report forms asking if trial participants would like to learn the trial results. This finding aligns with recommendations from researchers in other patient populations that emphasize the importance of early planning for results dissemination [7, 8, 10, 14, 29].

#### Plan to disclose treatment arm assignment upon request

One hundred and twenty (82.2%) survey respondents wished to know which antiseptic solution they received prior to surgery, which we subsequently communicated via email. While literature on this topic was limited, this rate is higher than in another study where 63.2% of interviewed mothers who participated in a clinical trial during their pregnancy wished to know which treatment arm they received [15]. Another trial on nutritional supplements and cataracts found slightly less than 79% of survey respondents accepted the offer to learn which treatment they received [26]. Researchers should anticipate that at least some of their participants may want to know their treatment arm upon learning the trial results and should have plans in place to share this information as requested.

## Conclusions

Given the low response rate and limited understanding from the results sharing in this study, future researchers should develop strategies to engage participants at every stage of the clinical trial to ensure they are upholding the principles outlined in the Declaration of Helsinki [12]. Discussing plans for sharing results with participants while they are actively participating in the initial trial could be beneficial and ameliorate many of the challenges faced in this study. By only contacting participants who explicitly wish to be informed of the findings, orthopaedic trauma researchers may avoid bringing up any unpleasant memories and having unnecessary contact with participants who indicated not wanting to be informed. Additionally, it is likely that our response rate may have improved had our participants been expecting to receive the PREPARE trial results. Given trial participants’ invaluable contribution to the field, orthopaedic trauma researchers should explore ways to better engage patients in the process of disseminating clinical trial findings and create plans for how results will be shared.

## Supplementary Information

Supplementary Material 1.

## Data Availability

The datasets used and/or analysed during the current study are available from the corresponding author on reasonable request.

## Abbreviations

CI: Confidence Interval
HHS: Hamilton Health Sciences
HiREB: Hamilton Integrated Research Ethics Board
REDCap: Research Electronic Data Capture
SDMs: Substitute Decision Makers
