# Supplementary figures and images for: Closing the loop: Benefits and challenges of sharing clinical trial results with participants after trial close-out
Source: BMC Med Res Methodol. 2026 Feb 4;26:56. doi: 10.1186/s12874-026-02787-3 (PMC12964888; doi:10.1186/s12874-026-02787-3)

**Appendix: Results Posters**

**
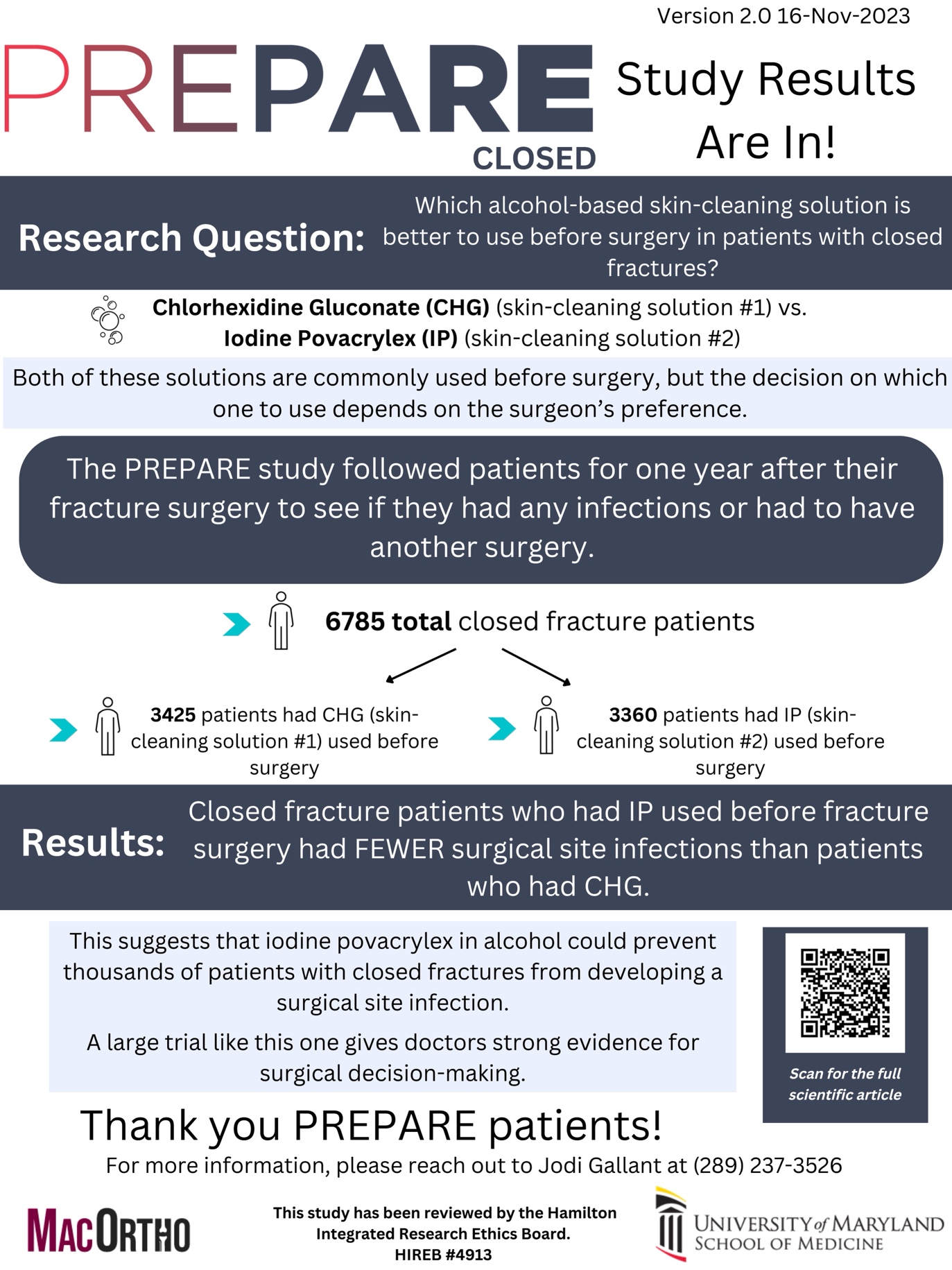
**

**
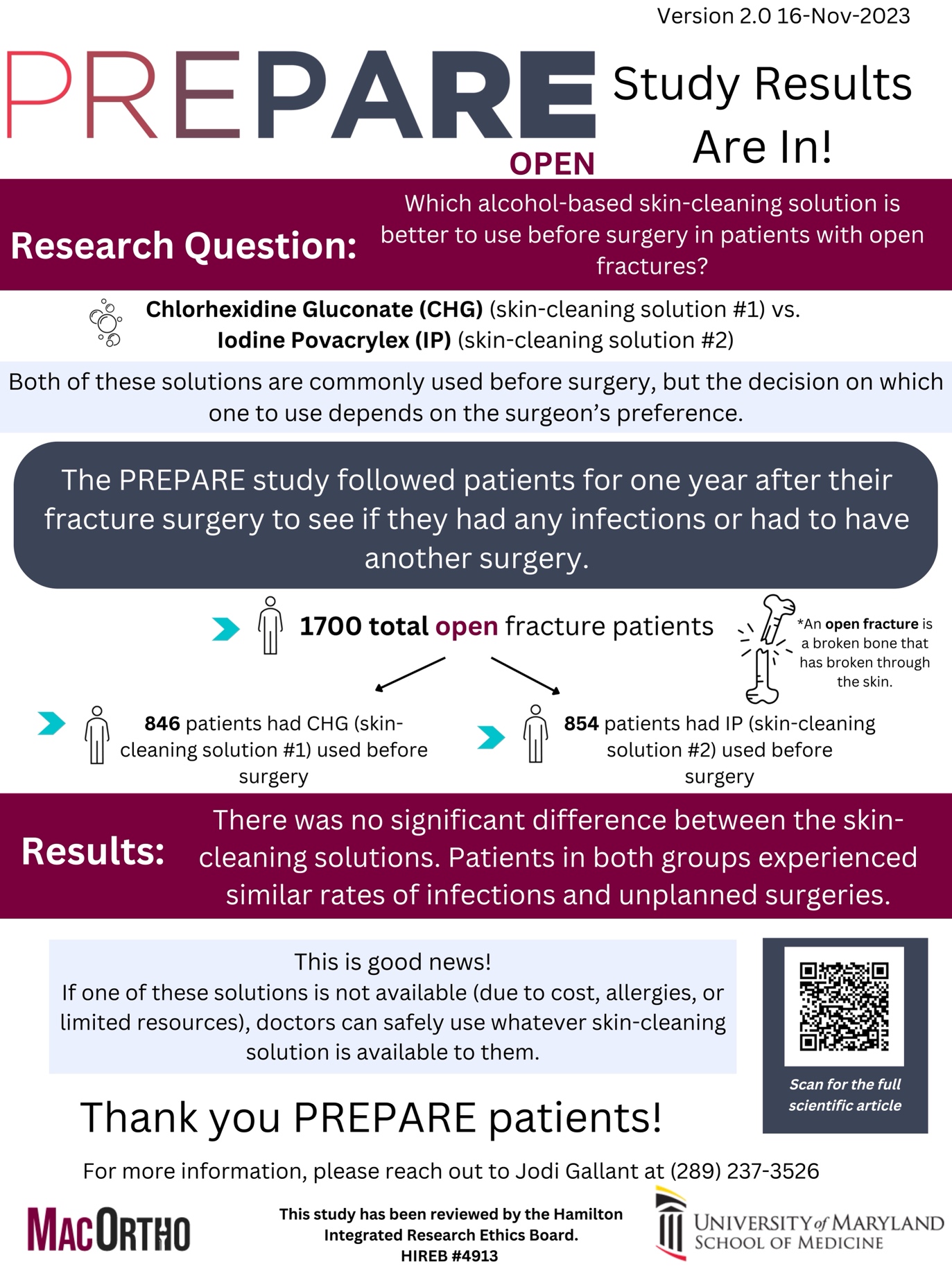
**

Supplement: Supplementary file 1 — Supplementary Material 1. [file 12874_2026_2787_MOESM1_ESM.docx]
